# Achieving a Textbook Outcome in Colon Cancer Surgery Is Associated with Improved Long-Term Survival

**DOI:** 10.3390/curroncol30030220

**Published:** 2023-02-28

**Authors:** Dimitrios K. Manatakis, Maria Tzardi, John Souglakos, John Tsiaoussis, Christos Agalianos, Ioannis D. Kyriazanos, George Pechlivanides, Athanasios Kordelas, Nikolaos Tasis, Nikolaos Gouvas, Evaghelos Xynos

**Affiliations:** 1 Medical School, University of Crete, 71003 Heraklion, Greece; 2 Department of Surgery, Athens Naval and Veterans Hospital, 11521 Athens, Greece; 3 Laboratory of Pathology, Medical School, University of Crete, 71003 Heraklion, Greece; 4 Laboratory of Translational Oncology, Medical School, University of Crete, 71003 Heraklion, Greece; 5 Laboratory of Anatomy, Medical School, University of Crete, 71003 Heraklion, Greece; 6 Department of Pathology, Athens Naval and Veterans Hospital, 11521 Athens, Greece; 7 Medical School, University of Cyprus, 99010 Nicosia, Cyprus; 8 Colorectal Unit, Creta Interclinic Hospital, 71304 Heraklion, Greece

**Keywords:** colon cancer, colon adenocarcinoma, textbook outcome, cancer survival, complete mesocolic excision

## Abstract

Background: Colon cancer surgery is a complex clinical pathway and traditional quality metrics may exhibit significant variability between hospitals and healthcare providers. The Textbook Outcome (TO) is a composite quality marker capturing the fraction of patients, in whom all desired short-term outcomes of care are realised. The aim of the present study was to assess the TO in a series of non-metastatic colon cancer patients treated with curative intent, with emphasis on long-term survival. Methods: Stage I–III colon cancer patients, who underwent curative colectomy following the Complete Mesocolic Excision principles, were retrospectively identified from the institutional database. TO was defined as (i) hospital survival, (ii) radical resection, (iii) no major complications, (iv) no reintervention, (v) no unplanned stoma and (vi) no prolonged hospital stay or readmission. Results: In total, 128 patients (male 61%, female 39%, mean age 70.7 ± 11.4 years) were included in the final analysis. Overall, 60.2% achieved a TO. The highest rates were observed for “hospital survival” and “no unplanned stoma” (96.9% and 97.7%), while the lowest rates were for “no major complications” and “no prolonged hospital stay” (69.5% and 75%). Older age, left-sided resections and pT4 tumours were factors limiting the chances of a TO. The 5-year overall and 5-year cancer-specific survival were significantly better in the TO versus non-TO subgroup (81% vs. 59%, *p* = 0.009, and 86% vs. 65%, *p* = 0.02, respectively). Conclusions: Outcomes in colon cancer surgery may be affected by patient-, doctor- and hospital-related factors. TO represents those patients who achieve the optimal perioperative results, and is furthermore associated with improved long-term cancer survival.

## 1. Introduction

The quality of surgical care has been the focus of healthcare systems for several decades. Various quality markers have been introduced, including postoperative mortality and morbidity. However, no single measure can capture the multifaceted aspect of the surgical pathway from the patients’ perspective, as outcomes may be affected by patient-, doctor- and hospital-related factors [1].

Composite outcome measures, on the other hand, may be more meaningful and clinically relevant [2,3,4,5,6]. They better reflect the multidimensional surgical practice, prevent indicator-driven practice and are a more appropriate summary of overall hospital performance [7]. Particularly for oncological patients, optimisation of cancer care pathways can be beneficial in terms of overall survival and quality of life. 

Textbook Outcome was first proposed for colon cancer patients in 2013 [8]. It is defined as receipt of optimal surgical care and represents the proportion of patients for whom all desired short-term outcomes of care are realised. However, evidence on the association of Textbook Outcome and long-term survival is limited.

The aim of the present study was to evaluate the colon cancer surgical pathway by assessing the Textbook Outcome in a cohort of non-metastatic colon cancer patients treated with curative intent, with emphasis on long-term survival.

## 2. Materials and Methods

Colorectal cancer patients treated at the Department of Surgery, Athens Naval and Veterans Hospital, between 2010 and 2020 were retrospectively identified from the institutional electronic database. Requirement for ethical approval was waived, as this was a retrospective, non-interventional study, and all data were anonymously analysed. 

Included in the analysis were all adult patients (age ≥ 18 years) with non-metastatic colon adenocarcinoma (stage I–III), who underwent colectomy with curative intent following the principles of Complete Mesocolic Excision (CME). Complete Mesocolic Excision involves (1) sharp dissection and mobilisation of the mesocolon along the embryological planes, (2) proximal resection margin > 10 cm, (3) distal resection margin > 5 cm, (4) central (high) vascular ligation of the main supplying vessels and (5) preservation of the integrity of the mesocolon [9]. Both elective and acute cases were included. Recurrent and metastatic cancers, as well as patients undergoing palliative or non-CME surgery, were excluded. Rectal cancers and histological types other than adenocarcinoma were also excluded, as they involve different treatment pathways and different outcome indicators. 

For each patient, the following data were collected: (i) demographic (age, sex, ASA class); (ii) intra-operative (tumour location, elective vs. emergency setting, open vs. laparoscopic approach, type of resection); (iii) immediate postoperative outcomes (30-day mortality, 30-day morbidity, length of stay—LOS, readmission, reoperation); (iv) histopathological (pT, pN, pTNM stage AJCC 8th edition, total lymph node yield, R status quality of resection); and (v) oncological data (length of follow-up, 5-year overall, cancer-specific and disease-free survival).

Textbook Outcome was assessed by 6 separate “desired outcome” measures, namely (1) hospital survival, (2) radical resection, (3) no major complications, (4) no reintervention, (5) no unplanned stoma and (6) no prolonged LOS or readmission [8]. These measures are ranked in decreasing order of importance, with hospital survival as the most important and LOS as the least important. Postoperative mortality was defined as mortality within 30 days after surgery. Radical resection was defined as microscopic radical resection (R0) plus a minimum of 12 lymph nodes in the specimen, as per national and international guidelines [10]. Postoperative morbidity was classified according to the Clavien–Dindo classification [11]. Major complications were defined as Clavien–Dindo II or higher, within 30 days after surgery. Reinterventions included either percutaneous procedures under local anaesthesia or reoperations under general anaesthesia. The desired LOS was set at the 75th percentile of the study population. Readmission was defined as unplanned readmission within 30 days following discharge.

Initially, the number and proportion of patients, for whom each individual outcome was reached, were calculated. A Textbook Outcome was attained when all 6 outcomes were reached. Clinical and histopathologic characteristics were then compared between the subgroups of patients who did or did not achieve the Textbook Outcome (group TO versus group NTO). Kaplan–Meier survival analysis was applied to calculate 5-year overall, cancer-specific and disease-free survival rates and to compare the two subgroups.

Continuous variables were expressed as mean ± standard deviation, while categorical variables were expressed as frequencies or percentages. Statistical analysis was performed on SPSS, version 20.0, using Student’s t-test for continuous variables and Chi-square or Fisher’s exact test for categorical variables. Statistical significance was set at *p* < 0.05.

## 3. Results

A total of 128 patients fulfilled the inclusion criteria and were included in the final analysis. Demographic and operative characteristics are shown in Table 1. The mean age of patients was 70.7 ± 11.4 years (range 36–93 years), with 61% being males and 39% females. The majority was elective cases (118, 92.2%), while 10 cases were emergencies (7.8%). The surgical approach was open in 78 (61%) and laparoscopic in 50 (39%). The conversion rate was 2.4% (3 cases). Regarding the type of resection, 58 patients underwent right hemicolectomy (45.3%), 19 patients extended right hemicolectomy (14.8%), 16 patients left hemicolectomy (12.5%) and 35 patients high anterior resection (27.3%).

Thirty-day post-operative mortality was 3.1% (4 cases). Overall, 30-day post-operative morbidity was 33.6% (43 cases), with major morbidity (Clavien–Dindo ≥ II) at 30.5% (39 cases). Mean LOS was 9.9 ± 7.7 days (median 7 days, range 3–49). For open resections, mean LOS was 10 ± 6.4 days, whereas for laparoscopic it was 9.7 ± 9.6 days. The 75th percentile was calculated at ≤11 days. Thirty-day readmission rate was 3.1% (4 cases). 

Pathology outcomes are shown in Table 2. Overall, 37 patients were classified as pTNM stage I (28.9%), 47 patients as stage II (36.7%) and 44 patients as stage III (34.4%). An R0 resection was attained in 124 cases (96.9%), with 4 cases being R1 (3.1%). These were all pT4N(+) cancers. The mean lymph node yield was 25.5 ± 11.4 (range 6–74 lymph nodes). One hundred and twenty-two patients had ≥12 lymph nodes in the surgical specimen (95.3%).

**Figure 1:**
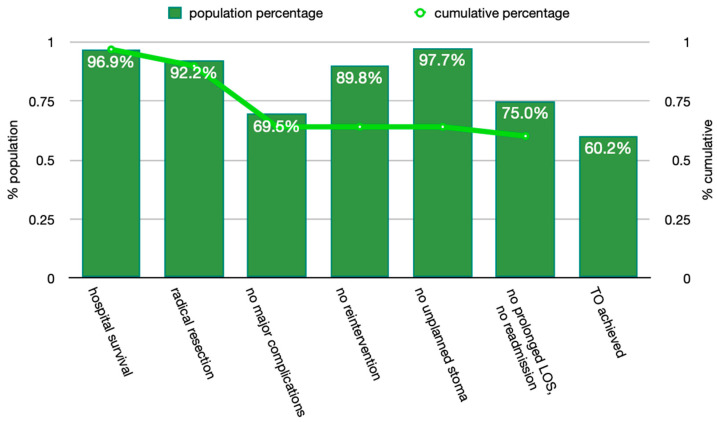
Textbook Outcome parameters (Bars depict the percentage of patients meeting each individual criterion. Line depicts the cumulative percentage of patients meeting each criterion, on condition that all previous criteria are also met).

Overall, 77 patients (60.2%) achieved the Textbook Outcome. Figure 1 shows the absolute percentage of patients for whom each desired outcome was realised, as well as the cumulative percentage, on the condition that all previous outcomes were achieved. The highest rates were observed for “no unplanned stoma” and “no mortality” (97.7% and 96.9%, respectively), whereas the lowest rates were for “no major complications” and “no prolonged hospital stay” (69.5% and 75%, respectively). When the two subgroups were compared, factors preventing Textbook Outcome were older age, left-sided and pT4 cancers (Table 3).

**Figure 2:**
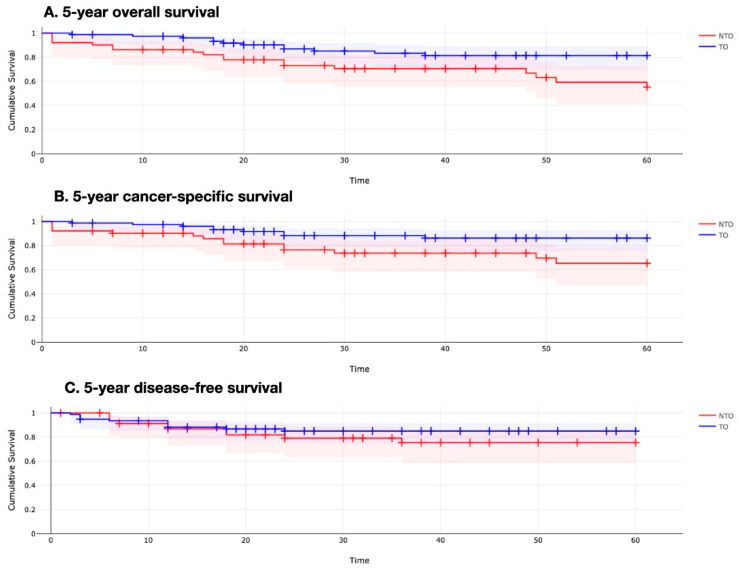
5-year survival curves between Textbook Outcome (TO) and Non-Textbook Outcome (NTO) patients.

Both 5-year overall and 5-year cancer-specific survival were significantly better in the Textbook Outcome subgroup (81% vs. 59%, *p* = 0.009, and 86% vs. 65%, *p* = 0.02, respectively). The 5-year disease-free survival showed a trend in favour of Textbook Outcome; however, this did not reach statistical significance (85% vs. 75%, *p* = 0.33) (Figure 2).

## 4. Discussion

The management of colon cancer is a complex, multidisciplinary pathway, involving colorectal surgeons, endoscopists, radiologists, pathologists and medical oncologists. Various quality indicators have been traditionally used to benchmark hospital performance regarding oncologic surgery, including, among others, postoperative mortality and morbidity, reoperation and readmission rates, lymph node yield and surgical specimen quality of resection, as well as cancer-related survival. However, these metrics may exhibit significant variability among hospitals and healthcare providers, as individual centres may score well in one indicator and poorly in another [12]. Moreover, some of these outcomes occur relatively infrequently to allow meaningful comparisons, whereas others may be poorly understood by the general public [12,13]. Patients, however, seek a more holistic approach to their perioperative journey and generally prefer summarising metrics of healthcare quality rather than more detailed single outcomes [6,7]. To overcome the weaknesses of single outcome measures, the Textbook Outcome was developed as a composite quality marker to capture the fraction of patients, in whom “everything goes well”. This is likely more meaningful and reflects what patients are most likely to value from their hospital experience [12].

Auditing the colon cancer treatment pathway in our department, the present study found that a Textbook Outcome was achieved in 60% of colon cancer patients treated with curative intent by the CME approach. The main limiting factors were major postoperative morbidity and prolonged hospital LOS. Overall Textbook Outcome rates in the colon cancer literature range between 49 and 67%, depending on the constituent variables. Similar to our results, most studies agree that postoperative complications are the main parameter reducing patients’ chances of a Textbook Outcome. While certain criteria are almost universally attained, for example “no postoperative mortality” (95–97%), “no major postoperative morbidity” ranges between 65 and 85% [1,8,13,14]. Indeed, this depends largely on the definition of what constitutes a severe postoperative complication. Studies so far have used a somewhat ambiguous terminology, for example “any adverse outcome within 30 days after resection” or “any postoperative surgical complication”. Our study used the standardised Clavien–Dindo classification and included grades ≥ II as major postoperative morbidity; although, this may be considered as a rather strict criterion. 

As expected, unplanned stoma rate was very low (2.3%), whereas reintervention (Clavien–Dindo grade IIIa—procedures under local anaesthesia or Clavien–Dindo grade IIIb—reoperation under general anaesthesia) was acceptable at 10.2%. On the other hand, hospital LOS depends, among other things, on the surgical approach; although, no statistical difference was observed between open and laparoscopic surgery. Further to that, there is no structured, pre-defined enhanced recovery protocol in our department, and implementation of any ERAS interventions is selective. 

Comparing TO versus NTO subgroups, older age, left-sided and locally advanced pT4 cancers limited chances of a Textbook Outcome. Overall TNM stage and pN stage marginally did not reach statistical significance, probably due to the sample size. Younger age, female sex, ASA II, elective surgery, right-sided resections and lower tumour stage are generally associated with increased chances of a Textbook Outcome [8]. Kolfschoten et al. found that 60% of the low-risk patients achieved a Textbook Outcome, compared to only 21% of the high-risk ones [8]. 

Importantly, 5-year overall and 5-year cancer-specific survival rates were significantly higher for Textbook Outcome patients. Yang et al. also reported better 5-year disease-specific survival (80% for TO versus 58% for NTO groups), whereas Aquina et al. found that every 10% increase in Textbook Oncologic Outcome rates was significantly associated with improved 5-year overall survival [1,15]. 

Healthcare services need to ensure that all colon cancer patients receive care which is safe, effective, patient-centred, timely, efficient and equitable [7]. Most of these parameters are reflected in the Textbook Outcome. Safety is measured by postoperative mortality and morbidity, efficiency by reintervention, readmission and hospital stay, whereas effectiveness is measured by adequate lymph node yield and negative resection margins [1,7]. 

This “all-or-none” approach of composite outcome markers is simple, comprehensive, and has been shown to be suitable when success depends on meeting all indicators, as is the case with perioperative cancer care [8]. Composite outcomes have the added advantage of occurring more frequently compared to individual parameters, for example postoperative mortality [13]. Moreover, they show the multidimensional complexity of surgical cancer pathways; they prevent indicator-driven practice and can be adjusted for differences in case-mix. Textbook Outcome summarises hospital/department performance, identifies inter-hospital variability and sets high standards for the ideal hospital [7,8]. Assessing nationwide hospital performance, the Dutch ColoRectal Audit (2013–2015) found Textbook Outcome rates among participating hospitals ranging between 22 and 85% for colon cancer surgery [13]. Interestingly, outlier hospitals were not determined by an excess of high-risk cases, as might have been the case with tertiary referral centres, but they underperformed for low-risk patients as well [8]. Identifying best-performing hospitals can provide valuable information regarding centralisation of care on the regional and national levels, and can be a starting point for quality improvement [6,13].

Furthermore, Textbook Outcome is meaningful to all stakeholders. For patients, it represents their chances for the most favourable outcome in a specific hospital. For healthcare providers, it provides information on how often treatment is successful and thus drives quality improvement. For hospital administrations, it summarises indicators on patient safety, effectiveness and efficiency of care; whereas, for policy makers, it may guide surveillance programmes [8]. Depending on the setting, the Textbook Outcome can be adjusted to include different parameters that pragmatically reflect the cancer care reality, e.g., at a local, regional or national level or even low-, middle- versus high-income countries [16,17].

Although composite indicators simplify complex information, their use is not without controversy [18]. To summarise multiple measures into a single indicator, the constituent variables should be valid quality markers and representative of the bigger picture [18]. It is also important to include outcomes which are relevant to all stakeholders, as the definition of “everything goes well” holds a very different meaning for patients, surgeons and hospital administrations [13,18]. Regarding statistical methodology, banding of continuous variables into nominal categories is occasionally inevitable; however, this approach reduces statistical power. Weighting of individual parameters may pose another problem [18]. Kolfschotten et al. ranked the six variables in decreasing order of significance, however without assigning weighting [8]. Therefore, postoperative mortality is statistically equally as important as hospital LOS. On the other hand, the interpretation of weighted results becomes more complicated, particularly for the general public [13]. To provide clear results, methodological transparency is key. Textbook Outcome should be presented with adequate technical information about the composite design and interpretation of results [18]. 

As far as the published studies to date are concerned, there is substantial variability in the definitions of Textbook Outcome. This can largely be attributed to the availability of data, for example, whether researchers used institutional databases or large national registries [12]. There is also inevitably a degree of overlap among the parameters used in constructing the Textbook Outcome [12]. Postoperative morbidity, for instance, is also reflected in reintervention rates, readmission rates and hospital LOS, and is thus captured multiple times across the constituent variables. When detailed morbidity data are available, inclusion of hospital LOS may be debatable [12]. Total LOS may not be important for most patients and is also affected by factors entirely outside the control of a hospital or a healthcare provider (e.g., ensuring adequate social support at discharge) [12]. An alternative definition for major postoperative morbidity could be “any complication leading to reintervention or reoperation (Clavien–Dindo ≥ III) or prolonging LOS”. Using the 75th percentile as the upper LOS limit means that the maximum achievable Textbook Outcome rate will be around 75%.

Our research meeting recommended using the original six variables by Kolfschoten et al., despite an argument on the definition of “major” morbidity, and that “unplanned stoma” is quite rare in colon cancer surgery. We finally decided on stricter criteria and included Clavien–Dindo grades ≥ II as “major “ complications, which defined “radical resection” as R0 microscopic resection *plus* ≥ 12 lymph nodes as per guidelines, and grouped prolonged LOS along with 30-day readmission. In any case, this highlights the need for a consensus on Textbook Outcome definition in colorectal surgery, as was the case with oesophageal and liver surgery [19,20]. However, even these Delphi meetings have not completely eliminated this overlapping. For example, both postoperative major morbidity and hospital readmission have been included, while it is obvious that any readmission following discharge is almost definitely due to postoperative complications. Generally, the main focus of these expert meetings is towards effectiveness (as expressed by radical resection) on one hand (negative margins and adequate lymph node yield in our paper), and safety and efficiency on the other (as expressed by postoperative mortality, major morbidity, reintervention, stoma, readmission). Yet, an experts-only consensus may introduce potential bias and does not necessarily capture the patients’ perspective. To reach broader agreement, this process should ideally include all stakeholders (patients, doctors, hospital administrations and policy-makers) [12]. 

Our study focused solely on the surgical colon cancer pathway, excluding adjuvant chemotherapy from the Textbook Outcome parameters. Despite this limitation and the sample size, all patients were treated by CME, ensuring a uniform high standard and an oncologically optimal surgical procedure, with en bloc complete removal of the respective mesocolon with all locoregional lymph nodes [10]. Our results generally parallel the low-risk cohort described by Kolfschoten et al. [8]. However, being a single-centre study, it cannot be determined whether this observation is attributed to the case-mix of our patients (ratio of low- versus high-risk cases). Furthermore, the last months of the study period were affected by the COVID-19 pandemic, which dramatically changed oncology care worldwide, causing significant delays in treatment and elective pathway modifications to counteract the effects of lock-down, staff shortages and reduced theatre capacity [21,22,23,24].

## 5. Conclusions

Surgery for colon cancer is a multifaceted, complex pathway and outcomes are affected by patient-, doctor- and hospital-related factors. The Textbook Outcome is a composite quality metric aiming to summarise all aspects of perioperative care, and reflects those patients in whom “everything goes well”. Sixty percent of our colon cancer patients achieved a Textbook Outcome, with the main limiting factors being postoperative morbidity and prolonged length of hospital stay. Importantly, attaining the Textbook Outcome was beneficial for long-term cancer survival. A wider consensus is required, however, as there is considerable variability in the individual parameters incorporated into the Textbook Outcome in colorectal studies published to date.

## Figures and Tables

**Table 1:** Demographic and operative characteristics.

	N	%
**Age (years)**	70.7 ± 11.4	range 36–93
**Sex**		
male	78	60.9%
female	50	39.1%
**ASA class**		
II	92	71.9%
III	36	28.1%
**Surgical setting**		
elective	118	92.2%
emergency	10	7.8%
**Surgical approach**		
open	78	60.9%
laparoscopic	47	36.7%
conversion	3	2.4%
**Type of resection**		
right hemicolectomy	58	45.3%
extended right	19	14.9%
left hemicolectomy	16	12.5%
high anterior resection	35	27.3%
**30-day post-operative mortality**	4	3.1%
**30-day post-operative morbidity**	43	33.6%
**Length of hospital stay (days)**	9.9 ± 7.7	range 3–49
**30-day readmission**	4	3.1%

**Table 2:** Histopathological outcomes.

	N	%
**Quality of resection**		
R0	124	96.9%
R1	4	3.1%
**Mean lymph node yield**	25.5 ± 11.4	range 6–74
≥12 lymph nodes	122	95.3%
**pT stage**		
pT1	22	17.2%
pT2	16	12.5%
pT3	73	57%
pT4	17	13.3%
**pN stage**		
pN0	84	65.6%
pN (+)	44	34.4%
pN1 (1–3 lymph nodes)	23	18%
pN2 (≥4 lymph nodes)	21	16.4%
**TNM stage**		
stage I	37	28.9%
stage II	47	36.7%
stage III	44	34.4%

**Table 3:** Comparison of Textbook Outcome versus non-Textbook Outcome subgroups.

	TO	NTO	*p* Value
**Age (years)**	68.5 ± 11.1	73.9 ± 11.3	**0.005**
**Sex**			
male	46 (59.7%)	32 (62.7%)	
female	31 (40.3%)	19 (37.3%)	0.733
**ASA class**			
II	57 (74%)	35 (68.6%)	
III	20 (26%)	16 (31.4%)	0.506
**Tumour location**			
right-sided	53 (68.8%)	24 (47.1%)	
left-sided	24 (31.2%)	27 (52.9%)	**0.013**
**Surgical setting**			
elective	73 (94.8%)	45 (88.2%)	
emergency	4 (5.2%)	6 (11.8%)	0.195
**Surgical approach**			
open + conversion	48 (62.3%)	33 (64.7%)	
laparoscopic	29 (37.7%)	18 (35.3%)	0.785
**TNM stage**			
stage I	28 (36.3%)	9 (17.6%)	
stage II	27 (35.1%)	20 (39.3%)	
stage III	22 (28.6%)	22 (43.1%)	0.056
**pT stage**			
pT1 + pT2	29 (37.7%)	9 (17.6%)	
pT3	42 (54.5%)	31 (60.8%)	
pT4	6 (7.8%)	11 (21.6%)	**0.012**
**pN stage**			
pN0	55 (71.4%)	29 (56.9%)	
pN(+)	22 (28.6%)	22 (43.1%)	0.089

## Data Availability

The data presented in this study are available on request from the corresponding author.

## References

[1] Yang C.C., Tian Y.F., Liu W.S., Chou C.L., Cheng L.C., Chu S.S., Lee C.C. (2020). The association between the composite quality measure “textbook outcome” and long term survival in operated colon cancer. Medicine.

[2] Busweiler L.A., Schouwenburg M.G., van Berge Henegouwen M.I., Kolfschoten N.E., de Jong P.C., Rozema T., Wijnhoven B.P., van Hillegersberg R., Wouters M.W., van Sandick J.W. (2017). Textbook outcome as a composite measure in oesophagogastric cancer surgery. Br. J. Surg..

[3] Oesophago-Gastric Anastomotic Audit (OGAA) Collaborative (2022). Textbook outcome following oesophagectomy for cancer: International cohort study. Br. J. Surg..

[4] Van Roessel S., Mackay T.M., van Dieren S., van der Schelling G.P., Nieuwenhuijs V.B., Bosscha K., van der Harst E., van Dam R.M., Liem M.S., Festen S. (2020). Textbook outcome: Nationwide analysis of a novel quality measure in pancreatic surgery. Ann. Surg..

[5] De Graaff M.R., Elfrink A.K., Buis C.I., Swijnenburg R.J., Erdmann J.I., Kazemier G., Verhoef C., Mieog J.S., Derksen W.J., van den Boezem P.B. (2022). Defining textbook outcome in liver surgery and assessment of hospital variation: A nationwide population-based study. Eur. J. Surg. Oncol..

[6] Sweigert P.J., Eguia E., Baker M.S., Link C.M., Hyer J.M., Paredes A.Z., Tsilimigras D.I., Husain S., Pawlik T.M. (2021). Assessment of cancer center variation in textbook oncologic outcomes following colectomy for adenocarcinoma. J. Gastrointest. Surg..

[7] Warps A.K., Detering R., Tollenaar R.A.E.M., Tanis P.J., Dekker J.W.T., Dutch C.A.G. (2021). Textbook outcome after rectal cancer surgery as a composite measure for quality of care: A population-based study. Eur. J. Surg. Oncol..

[8] Kolfschoten N.E., Kievit J., Gooiker G.A., Van Leersum N.J., Snijders H.S., Eddes E.H., Tollenaar R.A., Wouters M.W., Marang-Van De Mheen P.J. (2013). Focusing on desired outcomes of care after colon cancer resections; hospital variations in ‘textbook outcome’. Eur. J. Surg. Oncol..

[9] Hohenberger W., Weber K., Matzel K., Papadopoulos T., Merkel S. (2009). Standardized surgery for colonic cancer: Complete mesocolic excision and central ligation--technical notes and outcome. Colorectal Dis..

[10] Xynos E., Gouvas N., Triantopoulou C., Al E. (2016). Clinical practice guidelines for the surgical management of colon cancer: A consensus statement of the Hellenic and Cypriot Colorectal Cancer Study Group by the HESMO. Ann. Gastroenterol..

[11] Dindo D., Demartines N., Clavien P.A. (2004). Classification of surgical complications: A new proposal with evaluation in a cohort of 6336 patients and results of a survey. Ann. Surg..

[12] Aiken T., Abbott D.E. (2020). Textbook oncologic outcome: A promising summary metric of high-quality care, but are we on the same page. J. Surg. Oncol..

[13] van Groningen J.T., Ceyisakar I.E., Gietelink L., Henneman D., van der Harst E., Westerterp M., Marang-van de Mheen P.J., Tollenaar R.A., Lingsma H., Wouters M.W. (2020). Identifying best performing hospitals in colorectal cancer care; is it possible. Eur. J. Surg. Oncol..

[14] Mehta R., Tsilimigras D.I., Paredes A.Z., Sahara K., Moro A., Farooq A., White S., Ejaz A., Tsung A., Dillhoff M. (2020). Comparing textbook outcomes among patients undergoing surgery for cancer at US News & World Report ranked hospitals. J. Surg. Oncol..

[15] Aquina C.T., Hamad A., Becerra A.Z., Cloyd J.M., Tsung A., Pawlik T.M., Ejaz A. (2021). Is textbook oncologic outcome a valid hospital-quality metric after high-risk surgical oncology procedures. Ann. Surg. Oncol..

[16] Global*Surg* Collaborative and NIHR Global Health Research Unit on Global Surgery (2021). Global variation in postoperative and complications after cancer surgery: A multicentre, prospective cohort study in 82 countries. Lancet.

[17] Global*Surg* Collaborative and NIHR Global Health Research Unit on Global Surgery (2022). Effects of hospital facilities on patient outcomes after cancer surgery: An international, prospective, observational study. Lancet Glob. Health.

[18] Barclay M., Dixon-Woods M., Lyratzopoulos G. (2019). The problem with composite indicators. BMJ Qual. Saf..

[19] Kalff M.C., Van Berge Henegouwen M.I., Gisbertz S.S. (2021). Textbook outcome for esophageal cancer surgery: An international consensus-based update of a quality measure. Dis. Esophagus..

[20] Görgec B., Cacciaguerra A.B., Pawlik T.M., Aldrighetti L.A., Alseidi A.A., Cillo U., Kokudo N., Geller D.A., Wakabayashi G., Asbun H.J. (2022). An international expert delphi consensus on defining textbook outcome in liver surgery (TOLS). Ann. Surg..

[21] Glasbey J.C., Nepogodiev D., Simoes J.F., Omar O., Li E., Venn M.L., Abou Chaar M.K., Capizzi V., Chaudhry D., Desai A. (2021). Elective cancer surgery in COVID-19-free surgical pathways during the SARS-CoV-2pandemic: An international, multicenter, comparative cohort study. J. Clin. Oncol..

[22] Glasbey J.C., Nepogodiev D., Simoes J.F., Omar O.M., Venn M.L., Evans J.P., Futaba K., Knowles C.H., Minaya-Bravo A., Covid*Surg.* Collaborative (2020). Outcomes from elective colorectal cancer surgery during the SARS-CoV-2 pandemic. Colorectal Dis..

[23] Covid*Surg.* Collaborative (2021). Effect of COVID-19 pandemic lockdowns on planned cancer surgery for 15 tumour types in 61 countries: An international, prospective, cohort study. Lancet Oncol..

[24] Adamina M., Ademuyiwa A., Adisa A., Bhangu A.A., Bravo A.M., Cunha M.F., Emile S., Ghosh D., Glasbey J.C., Covid*Surg.* Collaborative (2022). The impact of surgical delay on resectability of colorectal cancer: An international prospective cohort study. Colorectal Dis..

